# Effect of the Addition of Acetylated Polysaccharides on the Properties of an Active Packaging Based on Polysuccinimide and Oregano Essential Oil

**DOI:** 10.3390/polym17212903

**Published:** 2025-10-30

**Authors:** Ignacio Antonio Hernández-Pérez, María Hernández-González, Alejandro Vega-Rios, América Chávez-Martínez, Ana Luisa Rentería-Monterrubio, Rogelio Sánchez-Vega, Ana Margarita Rodríguez-Hernández, Mario Alberto Morales-Ovando, Juan Manuel Tirado-Gallegos

**Affiliations:** 1 Facultad de Zootecnia y Ecología, Universidad Autónoma de Chihuahua, Periférico R, Almada km 1, Chihuahua 31453, Chihuahua, Mexico; p371866@uach.mx (I.A.H.-P.); amchavez@uach.mx (A.C.-M.); arenteria@uach.mx (A.L.R.-M.); rsanchez@uach.mx (R.S.-V.); 2 Departamento de Ciencia y Tecnología de Alimentos, División de Ciencia Animal, Universidad Autónoma Agraria Antonio Narro, Calzada Antonio Narro 1923, Buenavista, Saltillo 23515, Coahuila, Mexico; 3 Centro de Investigación en Materiales Avanzados, S.C. (CIMAV), Miguel de Cervantes #120, Complejo Industrial Chihuahua, Chihuahua 31136, Chihuahua, Mexico; alejandro.vega@cimav.edu.mx; 4 Centro de Investigación en Química Aplicada (CIQA), Enrique Reyna Hermosillo, No. 140, col. San José de los Cerritos, Saltillo 25294, Coahuila, Mexico; ana.rodriguez@ciqa.edu.mx; 5 Facultad de Ciencias de la Nutrición y Alimentos, Universidad de Ciencias y Artes de Chiapas, Libramiento Norte Poniente No. 1150, Tuxtla Gutiérrez 29039, Chiapas, Mexico; mario.morales@unicach.mx

**Keywords:** *Agave Lechuguilla* Torr, thermal properties, starch, mechanical properties, microcellulose

## Abstract

Polysuccinimide (PSI) is a biodegradable, extended-release polymer with great potential for developing active food packaging. In this study, we prepared PSI films functionalized with oregano essential oil (OEO, 3.5% *w*/*w*) and reinforced with acetylated polysaccharides (corn starch and microcellulose from *Agave Lechuguilla* Torr fibers) with different degrees of substitution (DS; 0.44–1.25) at a constant concentration (22% *w*/*w*). Tensile strength (0.86–1.34 MPa), elasticity modulus (0.96–1.65 MPa) and elongation at break (14.16–21.66%) increased (*p* < 0.05) with DS in the reinforcing materials. The moisture content and solubility decreased from 13.17% to 9.81% and from 45.64% to 38.75%, respectively. With increasing DS, water vapor permeability (WVP) decreased by up to 56.4% compared to the control film (unacetylated polysaccharides). The DS of the reinforcing materials did not affect the antioxidant activity. Antibacterial activity against *Escherichia coli* and *Staphylococcus aureus* revealed similar inhibition halos for both bacteria, regardless of the DS. Thermogravimetric and calorimetric analysis showed that reinforcing PSI films with acetylated materials improves thermal stability. The results of this research suggest that PSI, a polymer derived from the thermal polymerization of aspartic acid, has significant potential for the development of eco-friendly active packaging for food products.

## 1. Introduction

Packaging plays a crucial role in food preservation by protecting it from environmental factors, facilitating its handling, and enhancing its marketing [1,2]. For this reason, the food industry requires large quantities of packaging, and demand for it is constantly increasing as society’s food preparation and consumption habits change [3,4,5]. Traditionally, food packaging is made from petroleum-derived synthetic polymers, as these materials offer various advantages, including good mechanical properties and low or zero permeability to oxygen and water vapor [6,7,8]. However, synthetic packaging is not biodegradable, and its accumulation has caused excessive environmental pollution [9]. Therefore, there is currently great interest in developing biodegradable packaging made from natural biopolymers, such as polysaccharides, in combination with other natural components, as an alternative to synthetic materials [10,11].

In this regard, one biodegradable polymer with potential for packaging applications is polysuccinimide (PSI), which is obtained by polycondensation of aspartic acid [12]. This non-toxic, biodegradable, and biocompatible material exhibits prolonged compound release, a property that has been studied in the pharmaceutical field to prolong the therapeutic effect of various drugs [13,14]. For this reason, it is necessary to incorporate plasticizing agents such as glycerol, which is naturally sourced and does not have adverse environmental effects. In addition, it reduces contact between polymer chains, thereby increasing their mobility and decreasing their rigidity [15]. On the other hand, natural polymers such as microcellulose and starch have been added to enhance their mechanical properties [16,17]. However, one limitation of these polysaccharides is their hygroscopic nature, attributed to the large number of hydroxyl groups (OH) in their structures [18]. The hydrophilic nature of these materials can be reduced through various chemical modifications [19,20]. Among these modifications, acetylation is one of the most widely used methods to reduce the hydrophilicity of polysaccharides [19]. This modification involves replacing the OH groups on glucose residues with hydrophobic acetyl groups (CH_3_CO) by reacting the polysaccharide with acetic acid, acetic anhydride, and sulfuric acid as a catalyst at different reaction times and temperatures [21]. To measure the effectiveness of the acetylation process, the degree of substitution (DS) is calculated in the product. The DS indicates the average number of OH groups substituted by glucose residues from the polysaccharide [19].

In addition to developing biodegradable packaging, the current trend focuses on active food packaging, which incorporates additives that can trap oxygen, release or absorb odors and flavors, and provide antioxidant and antimicrobial properties [2,22]. The current trend is to use natural additives, particularly essential oils (EO) extracted from plants and spices, as potential active agents due to their functional properties; however, their application requires evaluating factors such as concentration and their effect on the sensory properties of foods [23,24]. EOs are an abundant source of biologically active compounds, including flavonoids, terpenoids, and phenolic acids [25]. They are used in the food industry due to their antioxidant and antimicrobial properties, which slow down or inhibit chemical oxidation reactions and microbial growth [2,26]. Furthermore, due to their hydrophobic nature, the incorporation of EOs can improve barrier properties by reducing water vapor permeability [26].

There are reports on the use of many EOs in the development of active, biodegradable food packaging; however, cinnamon, clove and oregano EOs stand out, which are mainly added to polysaccharide-based matrices [27]. Oregano essential oil (OEO) is primarily composed of thymol and carvacrol, both of which exhibit antifungal and antibacterial properties [25,28]. Thymol also stands out for its antioxidant activity [29]. OEO is usually used at higher concentrations in the formulation of food packaging than as a food additive. To assess the potential toxic effects of OEO, Llana-Ruiz-Cabello et al. [24] evaluated the subchronic toxicity of OEO administered orally at different doses (50, 100, and 200 mg/kg b.w./day) in Wistar rats for 90 days. These authors observed no mortality or significant effects on body weight during the study. No significant effect was observed on biochemical or haematological parameters in rats fed an OEO diet or in the control group (OEO-free diet). This study concludes that OEO can be used as an active agent in food packaging, as no toxicity was observed at levels up to 330 times higher than those to which consumers might be exposed.

In addition, when EOs are added to film formulations, they become trapped (encapsulated) within the polymer matrix, thereby decreasing their volatility and improving their efficiency [30]. Additionally, the architecture of the polymer matrices enables the controlled and sustained release of EOs from the film into the food [5]. In a study conducted by Hernández-González et al. [31], the PSI was functionalized with OEO at 3, 7 and 10% (*w*/*w*). The authors observed that PSI functionalized with 3% (*w*/*w*) OEO exhibited inhibition halos of 4.10 cm and 4.0 cm for *Escherichia coli* and *Staphylococcus aureus*, respectively. When the OEO concentration exceeded 3% (*w*/*w*), no significant differences were observed. Additionally, the migration study revealed that polysuccinamide is a sustained-release material. Therefore, the objective of this work was to obtain and characterize the properties of an active packaging based on PSI and OEO reinforced with MCC and starch with different DS.

## 2. Materials and Methods

### 2.1. Materials

The lechuguilla (*Agave Lechuguilla* Torr) fibers were provided by the Department of Food Science and Technology at the Universidad Autónoma Agraria Antonio Narro (UAAAN), Saltillo, Coahuila, Mexico. The PSI was obtained through the thermal polycondensation of *L*-aspartic acid (Sigma Aldrich, Toluca, Estado de México, México) at 250–300 °C, at which point a pinkish hue appeared [12]. The OEO was obtained by steam distillation, and its main components were thymol and carvacrol at a ratio of 1:4 [28]. Native corn starch was purchased at a local supermarket.

### 2.2. Acetylation of Polysaccharides

The microcellulose (MCC) was obtained from lechuguilla fibers following the methodology reported by Muhamad et al. [32]. The obtained MCC was acetylated according to the methods reported by Ávila Ramírez et al. [33] and Dewi et al. [34], with slight modifications. First, a dispersion of MCC (65% *w*/*v*) in glacial acetic acid was prepared in a flat-bottom flask. The mixture was heated for 1 h, and 0.47 g of tartaric acid per g of MCC was added, followed by stirring for 45 min. After this time, 2 mL of acetic anhydride per gram of MCC was added, and the flask was placed in an oil bath, connected to a glass condenser with water reflux. The mixture was heated to 120 ± 5 °C for different reaction times (1, 3 and 5 h) to obtain acetylated MCC with different DS. Subsequently, the samples were washed with distilled water until a pH of 6–7. Finally, the acetylated MCC was dried at 50 °C for 12 h and sieved through a 100-mesh sieve.

On the other hand, the acetylation of commercial corn starch was carried out according to the methodology proposed by Xu et al. [35], with slight modifications. A 20% (*w*/*v*) starch dispersion was prepared in acetic anhydride and stirred for 5 min. Next, 0.15 g of a 50% (*w*/*v*) sodium hydroxide (NaOH) solution was added to each gram of starch, and the mixture was heated to 120 ± 5 °C for 1, 2 and 3 h to obtain different DS. Subsequently, the acetylated starch was washed twice with distilled water and twice with 96% ethanol (*v*/*v*), alternating between water and ethanol washes. Finally, the acetylated starch was dried at 50 °C for 24 h and then ground in a mortar until it passed through a 100-mesh sieve. The DS and percentage of acetyl groups (*%Acetyl*) of the acetylated materials were calculated using the following equations:

(1) $$\begin{array}{c} \%Acetyl=\frac{\left(V_{b}-V_{s}\right)\times N\times 0.043}{W_{s}}\times 100 \end{array}$$

(2) $$\begin{array}{c} DS=\frac{162\times \%\mathrm{Acetyl}}{4300-(42\times \%\mathrm{Acetyl})} \end{array}$$

where *V_b_* is the volume (mL) of hydrochloric acid (HCl) used in the blanck, *V_s_* is the volume (mL) of HCl used in the sample, *N* is the normality of the acid, 0.043 is the milliequivalents of the acetyl group, *W_s_* is the weight (g) of the dried sample, 162 is the molecular weight of anhydroglucose and 4300 is the molecular weight of the acetyl group multiplied by 100.

### 2.3. Preparation of the Films

In Table 1, we describe the formulations for the four films evaluated in this research. The films were formulated according to previously conducted experimental studies by our research group [36]. Initially, the solid materials (MCC, starch and PSI) were mixed for 3 min in a 1 L beaker using a metal spatula. Next, OEO and glycerol were mixed for 3 min, as with the solids. After this, the solids and liquids were mixed for 5 min. The percentage of polysaccharides (22%) was a 1:1 mixture (MCC: starch), resulting in four treatments: film T1 (control treatment) presented a mixture of starch and MCC without acetylation, film T2 included a mix of starch and MCC acetylated for 1 h, film T3 contained starch acetylated for 2 h and MCC acetylated for 3 h, and finally, film T4 contained starch acetylated for 3 h and MCC acetylated for 5 h.

Extrusion of the materials was carried out in the extruder micro compounder (Xplore IM 15, Sittard, The Netherlands) at 125 °C with a screw speed of 60 rpm for a residence time of 3 min. During each load, 15 g of mixture was added, maintaining an internal force of 2000–2500 N to ensure consistent extrusion conditions across all treatments. After extrusion, 38 g of each material was deposited on 15 × 15 cm steel plates for molding in a thermoforming machine (PHI model Q230M, Los Angeles, CA, USA) at a pressure of 25 ton for 5 min at 150 °C. After this time, it was transferred to a cooling plate for 10 min at the same pressure (25 ton) under circulating water at room temperature. Subsequently, the films were conditioned for 48 h in a desiccator containing a saturated solution of sodium bromide (NaBr) (RH = 55% ± 5%). Finally, after conditioning, the properties described below were evaluated.

### 2.4. Molecular Properties

The study was conducted using Fourier transform infrared spectroscopy (FTIR) in the region of 4000–500 cm^−1^ with a spectrometer (model Spectrum Two, PerkinElmer Inc., Bucks, UK) equipped with an attenuated total reflectance (ATR) module. All samples were analyzed in triplicate under a puncture force of 80 N ± 1. The vibrational transition frequencies were recorded in transmittance (%), averaging 34 scans per sample and a resolution of 4 cm^−1^ [37].

### 2.5. Color

Color analysis was determined using a colorimeter (Konica Minolta Sensing CR 400, Osaka, Japan), where the variables of lightness (L*), coordinate a* (values +: red, values −: green) and coordinate b* (values +: yellow, values −: blue) were measured according to the CIELAB scale. Finally, the color difference (ΔE*) between the treatments and the control was calculated according to the following equation [38]:

(3) $$\begin{array}{c} ∆E*=\sqrt{{\left(∆a\right)}^{2}+{\left(∆b\right)}^{2}+{\left(∆L\right)}^{2}} \end{array}$$

where *Δa*, *Δb* and *ΔL* are the differences in the a* coordinate, the b* coordinate and the L* brightness of the films with acetylated materials and the control, respectively. The film without acetylated materials was used as the control. All readings were taken at five random points on the surface of the films.

### 2.6. Scanning Electron Microscopy

Morphological analysis was performed using an electron microscope (Hitachi SU3500, Tokyo, Japan) at 10 kV. The samples were fractured (under nitrogen) to examine the cross-section, and subsequently coated with a layer of gold.

### 2.7. Moisture Content, Water Solubility, and Contact Angle

The moisture content of the films was determined using the standard method of the International Association of Official Analytical Chemists (AOAC). Samples measuring 2 × 3 cm were cut, and their weights were recorded. The samples were then dried in a drying oven (SHEL LAB 1330 GM, Sheldon Manufacturing, Inc., Cornelius, OR, USA) at 105 °C for 2 h. This analysis was performed five times for each treatment, and the water content in the films was reported as a percentage of moisture according to the following equation:

(4) $$\begin{array}{c} Moisture(\%)=\frac{W_{i}-W_{f}}{W_{i}}\times 100 \end{array}$$

where *W_i_* is the initial weight of the wet sample and *W_f_* is the weight of the sample after drying.

The water solubility was determined according to the methodology described by Domene-López et al. [7]. For this test, the samples used in the moisture analysis were placed in beakers containing 80 mL of distilled water and stirred at 50 rpm on a stir plate (JOANLAB AC110, Huzhou, China) at room temperature for 24 h. Subsequently, the solids were recovered by filtration through previously dried Whatman No. 1 paper at 105 °C for 2 h. The residues from each sample were dried together with the filter paper at 105 °C until a constant weight was reached. This analysis was carried out five times, and the solubility percentage (%S) was determined using the following equation:

(5) $$\begin{array}{c} \%S=\frac{W_{i}-W_{f}}{W_{i}}\times 100 \end{array}$$

where *W_i_* is the initial weight of the dry film and *W_f_* is the final weight of the dry film after immersion in water for 24 h.

The contact angle of the films was determined using an FTA1000 contact angle tensiometer (First Ten Anstroms, Inc., Portsmouth, VA, USA). Five drops of distilled water were deposited on different regions of the surface of each film. After 60 s, the image was captured and analyzed with Fta32 video 2.0 Software FTA32 Software (First Ten Anstroms, Inc., Portsmouth, VA, USA) [39].

### 2.8. Thickness and Water Vapor Permeability (WVP)

Film thickness was measured using a Mitutoyo digital micrometer (Coolant Proof 293–298, Kawasaki, Kanagawa, Japan) at 10 random points on the films. Five films were used for each treatment in this measurement.

The WVP was evaluated according to the ASTM-E-96-80 [40] method, as described by Tirado-Gallegos et al. [37], known as the cup or test cell method. On the other hand, WVP was evaluated according to the guidelines of Method 3, as described by Manuel, which is known as the cup or test cell method. Samples with a radius of 2.5 cm were cut out and placed on acrylic cells containing 5 g of dehydrated silica gel as a desiccant (RH ≈ 0%). The cells were then placed in a desiccator containing a saturated sodium chloride (NaCl) solution to generate a relative humidity of 75%. The change in weight in the cell (*Δw*) with respect to time (*Δt*) was recorded every 60 min for 6 h. The recorded data were fitted to a straight line using linear regression, and the slope of the line (g·s^−1^) and the effective permeation area (m^2^) were used to obtain the water vapor transmission rate (*TR*) and *WVP* (g·Pa^−1^·s^−1^·m^−1^) using the following equations:

(6) $$\begin{array}{c} TR=\left(\frac{∆w}{∆t}\right)\frac{1}{A} \end{array}$$

(7) $$\begin{array}{c} WVP=\frac{\left(TR\right)\left(e\right)}{∆P} \end{array}$$

where *A* is the effective permeation area (m^2^); *e* is the film thickness (m); *ΔP* is the partial water vapor pressure gradient (Pa) in the desiccator and inside the cell.

### 2.9. Mechanical Properties

The mechanical properties of tensile strength (TS) in MPa, elastic modulus (EM) in MPa and elongation at break (%E) in % were determined under the guidelines of the ASTM-882-95a [41] standard. For each treatment, ten strips of film (10 mm × 60 mm) were cut, and their thickness was measured at 10 random points along each strip using a Mitutoyo digital micrometer (model Coolant Proof 293–298, Kawasaki, Kanagawa, Japan). The samples were subjected to tensile stress using a TAXT-Plus Estable Mycro Sistems texturer (Estable Mycro Sistems, TA-XT plus, Surrey, UK), with a 30 kg load cell, at a speed of 0.50 mm/s.

### 2.10. Thermal Properties

The thermal properties were evaluated using thermogravimetric analysis (TGA) with a TGA 5500 instrument (TA Instruments, New Castle, DE, USA). Samples of 4–6 mg from each treatment were weighed and heated from 50 to 700 °C with a heating ramp of 20 °C/min under a nitrogen atmosphere with a flow rate of 10 mL/min. The following thermal parameters were obtained from the thermogravimetric derivative (TGD) curves: the percentage of moisture content at 100 °C (MC100), the temperature at which a 10% weight loss occurs (T_90_), the temperature at which weight loss begins (T_onset_) and the residual mass at 700 °C (MR700) [37].

On the other hand, differential scanning calorimetry (DSC) analysis was performed in a DSC 2500 calorimeter (TA Instruments, New Castle, DE, USA). For this purpose, 5 mg of the sample was weighed and placed in an aluminum pan that was hermetically sealed; an empty pan was used as a reference. The samples were heated from 25 to 350 °C at 10 °C/min under an inert nitrogen atmosphere (70 mL/min). Three replicates were performed for each sample analyzed.

### 2.11. Antioxidant Activity

To assess antioxidant activity, extracts were obtained using the methodology reported by Martucci et al. [42], with slight modifications. Samples of 1 g for each treatment were mixed with 15 mL methanol and stirred for 24 h on a stirrer plate at 50 rpm. After this time, the samples were centrifuged at 5000× *g* for 30 min and then filtered through Whatman No. 1 paper. The recovered supernatants were used to evaluate their antioxidant activity against DPPH and ABTS radicals.

Antioxidant activity against the DPPH radical was evaluated following the methodology reported by Thaipong et al. [43], with minor adjustments. First, a solution (100 mL) of 0.06 mM DPPH (2,2-diphenyl-1-picrylhydrazyl) in methanol was prepared. From this solution, 10 mL was taken and mixed with 45 mL of methanol. The absorbance of this working solution was adjusted to 1.1 ± 0.02 at 515 nm. Finally, 20 μL of each sample was mixed with 280 μL of the DPPH working solution, and the absorbance was read at 515 nm. Likewise, a standard curve was created using Trolox (6-hydroxy-2,5,7,8-tetramethylchroman-2-carboxylic acid), and the antioxidant capacity was reported as mg Trolox equivalent per 100 g of dry sample (mg TE/100 g sample).

Antioxidant activity using the ABTS method described by Thaipong et al. [43], with slight modifications. A solution (10 mL) of 7.7 mM ABTS (2,20-azino-bis (3-ethylbenzthiazolin)-6-sulphonic acid) was prepared and mixed with a solution (10 mL) of 2.6 mM potassium persulfate, both prepared using distilled water. The mixture was left to stand in the dark for 12 h to generate the ABTS radical. Subsequently, the working solution was prepared by mixing 1 mL of the ABTS radical solution with 60 mL of methanol, and adjusting the absorbance to 1.1 ± 0.02 at 734 nm. Finally, 20 μL of each extract was mixed with 280 μL of the ABTS working solution and the absorbance was immediately read at 734 nm. Antioxidant activity was reported as mg Trolox equivalent per 100 g of dry sample (mg TE/100 g sample). All analyses were performed in triplicate, and readings were obtained using a Multiskan Go microplate reader (Thermo Scientific, Vantaa, Finland).

### 2.12. Antibacterial Activity

Antibacterial activity was determined using the disk diffusion method described by Pandey et al. [44], with slight modifications. *Escherichia coli* and *Staphylococcus aureus* were used as representatives of Gram-negative and Gram-positive microorganisms, respectively. For each microorganism, a culture was prepared at a concentration of 1 × 10^8^ colony-forming units per milliliter (CFU/mL). Circular samples were cut from the films using a 5 mm diameter hole punch. Subsequently, 100 μL of each microbial culture was taken and distributed on nutrient agar plates. A circle from each treatment was then placed on each plate. Moreover, a positive control containing 3.5% (*w*/*w*) glycerol and OEO was used. The plates were then incubated for 24 h at 37 °C in an incubator. Microbial activity was measured by determining the inhibition zones formed, including the diameter of the film sample. Five replicates were performed for each treatment.

### 2.13. Experimental Design

Variables were analyzed using analysis of variance (ANOVA) with the GLM procedure in SAS version 9.4, followed by a Tukey mean comparison test (*p* < 0.05) under a completely randomized design. The treatments analyzed are described in Table 1.

## 3. Results and Discussion

### 3.1. Percentage of Acetylation and Degree of Substitution

The percentages of acetyl groups and the degree of substitution for MCC of lechuguilla fibers and commercial corn starch were measured to evaluate the effectiveness of the acetylation process. As expected, the %Acetyl and DS values in the MCC and starch showed similar behavior, increasing significantly with reaction time (*p* < 0.05). The %Acetyl and DS values of acetylated microcellulose at different times were: 1 h (12.02 ± 0.02 %Acetyl; 0.52 ± 0.00 DS), 3 h (18.42 ± 0.11 %Acetyl; 0.85 ± 0.01 DS) and 5 h (22.31 ± 0.03 %Acetyl; 1.06 ± 0.01 DS). These values were within the range reported by Cheng et al. [19] (from 0.21 to 2.27 DS) and Dong et al. [45] (from 0.55 to 1.64 DS). Some authors report on the production of biodegradable packaging from acetylated cellulose with a DS of up to 2.5 [46,47]. Regarding corn starch, the following results were observed at different acetylation times: 1 h (10.65 ± 0.12 %Acetyl; 0.44 ± 0.01 DS), 2 h (15.61 ± 0.11 %Acetyl; 0.69 ± 0.01 DS) y 3 h (25.11 ± 0.27 %Acetyl; 1.25 ± 0.02 DS). The DS values are influenced by the type of polysaccharide, the acetylation method, the reagents, and the reaction conditions (temperature and time). In the case of starch, low values (0–0.2 DS) can be used as food additives, while medium values (0.2–1.5 DS) and high values (1.5–3.0 DS) can be used for packaging design [48].

### 3.2. Molecular Properties

**Figure 1:**
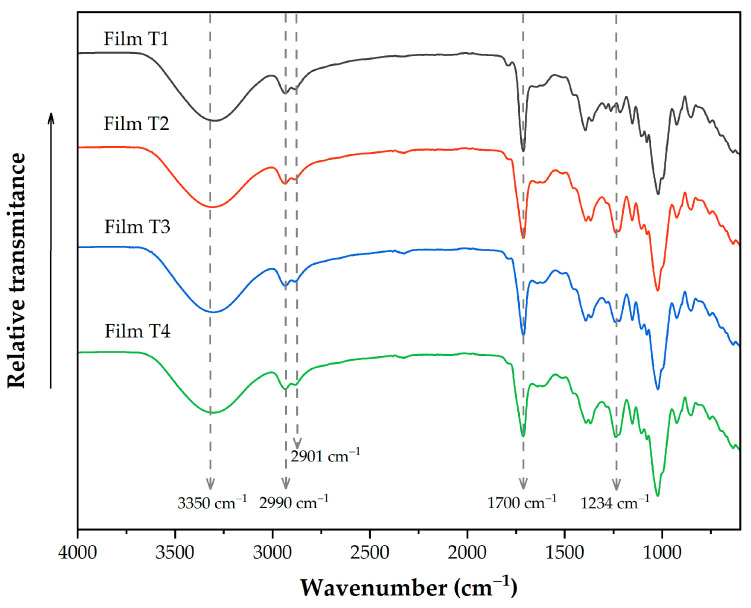
FTIR spectra of films produced with PSI, OEO, and acetylated polysaccharides. Film T1 = film with non-acetylated polysaccharides; Film T2 = film with MCC acetylated for 1 h and starch acetylated for 1 h; Film T3 = film with MCC acetylated for 3 h and starch acetylated for 2 h; Film T4 = film with MCC acetylated for 5 h and starch acetylated for 3 h.

Figure 1 shows the infrared spectra of the evaluated films, which reveal the most representative bands of the different functional groups present in these films. The band located around 3300 cm^−1^ corresponds to the stretching vibration of the OH groups present in glycerol and polysaccharides. As the acetylation time increased, the band intensity decreased; this behavior can be attributed to a lower concentration of OH groups in films containing acetylated polysaccharides [39]. The bands at 2990 and 2901 cm^−1^ are presented immediately, corresponding to the vibrations of the CH_2_ groups in microcellulose [3]. It overlaps with the C–H stretching vibration of the aromatic groups of OEO [49]. The band at 1700 cm^−1^ corresponds to carbonyl groups (C=O) present in PSI and acetylated polysaccharides [22,34]. It overlaps with the band at 1735 cm^−1^, related to the phenolic ring groups (–C_6_H_6_) [28]. The band observed around 1234 cm^−1^ in Films T2–T4 corresponds to the stretching C-O of the acetyl group. As the acetylation time increases, the intensity of this band also increases. In addition, this band is not present in film without acetylated reinforcement materials (Film T1), indicating that the elaboration process did not affect the presence of acetyl groups [3,50].

### 3.3. Moisture, Solubility, and Contact Angle

The moisture content and solubility percentage of the films produced are shown in Table 2. The trend in both variables was similar. Film T2 showed no significant differences compared with the control (Film T1). In contrast, films with reinforcing materials having a higher DS (Films T3 and T4) exhibited lower solubility and moisture content than Films T1 and T2 (*p* < 0.05). This behavior was attributed to decreases in OH groups and increases in acetyl groups in starch and MCC, which increased the films’ hydrophobicity [3]. These results are in agreement with those reported by El Halal et al. [51], who observed that the moisture content in starch films reinforced with cellulose fibers (20%, *w*/*w*) decreased from 16.55 to 12.78% when native starch in the formulation was replaced with acetylated starch (0.08 DS).

Regarding solubility in water, the results reported in Table 2 show behavior opposite to that reported by other authors, who have observed that starch acetylation promotes greater solubility of starch-based films [3,52,53]. In this case, water molecules can more easily enter the polymer matrix’s structure due to the low retrogradation [3]. Additionally, partial hydrolysis of starch during acetylation can yield shorter and more soluble glucose chains, thereby enhancing the solubility of polymer matrices [51]. On the other hand, reports indicate that an increase in DS values decreases film solubility, as reported by Schmidt et al. [54]. These authors observed solubility values of 25.68%, 20.31% and 21.62% in films prepared from native cassava starch, acetylated cassava starch with 0.6 DS, and acetylated cassava starch with 1.1 DS, respectively. According to the authors, solubility decreases due to a lower number of hydroxyl groups in acetylated starches.

**Figure 2:**
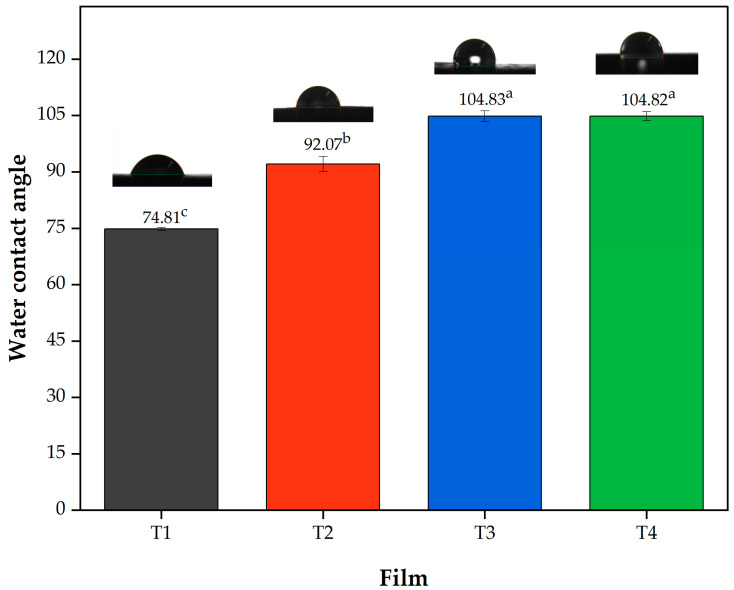
Contact angle values of the films produced with PSI, OEO, and acetylated polysaccharides. Means with different superscript letters (a–c) in the same column indicate statistically significant differences (*p* < 0.05). Film T1 = film with non-acetylated polysaccharides; Film T2 = film with MCC acetylated for 1 h and starch acetylated for 1 h; Film T3 = film with MCC acetylated for 3 h and starch acetylated for 2 h; Film T4 = film with MCC acetylated for 5 h and starch acetylated for 3 h.

Regarding the contact angle (Figure 2) it can be observed that the film T1 showed a lower angle (74.80 ± 0.35) being statistically different (*p* < 0.05) with respect to the other treatments, followed by Film T2 with values of 92.06 ± 2.03° (*p* < 0.05) and finally Films T3 and T4, which did not show statistically significant differences (*p* > 0.05) between them. These results can be attributed to the hydrophobicity of acetylated polysaccharides [55]. These results are consistent with the moisture content and water solubility (Table 2), which decreased with increasing DS, showing a lower affinity for water. Finally, according to Jiménez-Regalado et al. [3], contact angles above 90° indicate hydrophobicity; thus, films T2, T3 and T4 meet this criterion.

### 3.4. Color Properties

**Figure 3:**
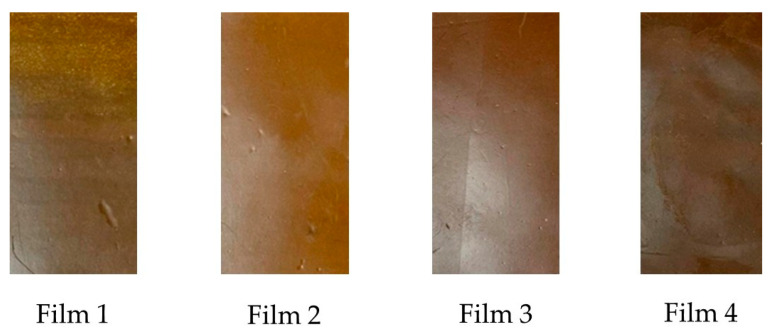
Photographic images of the films produced with PSI, OEO, and acetylated polysaccharides. Film T1 = film with non-acetylated polysaccharides; Film T2 = film with MCC acetylated for 1 h and starch acetylated for 1 h; Film T3 = film with MCC acetylated for 3 h and starch acetylated for 2 h; Film T4 = film with MCC acetylated for 5 h and starch acetylated for 3 h.

Packaging color is crucial, as it is the first contact consumers have with packaged products. The films evaluated in this study were prepared with materials of different colors. Table 2 presents the color variables in CIELAB (L*, a*, b*) space, along with the color difference (ΔE*), for all treatments. The films evaluated showed statistical variations in their optical properties, which were a consequence of the acetylation time of the polysaccharides added in their formulation. Films T1 and T2 presented the same values in their color variables L* and a*. However, variable b* was higher in Film T2, resulting in greater yellowing. In general, the b* variable increased with acetylation time across all treatments (*p* < 0.05), ranging from 4.34 to 13.24. Concerning the a* coordinate, its general trend was to decrease with increasing acetylation time of the reinforcing materials. Films T1 and T2 had the lowest value (9.21 ± 0.21), while the highest values were observed in Films T3 (11.65 ± 0.03) and T4 (17.17 ± 0.32), with significant differences between these two films, resulting in reddish materials (values + a*). On the other hand, the brightness L* decreased with the increase in the acetylation time of the reinforcing materials, such that Films T1 and T2 had the highest value (60.24 ± 0.28). In contrast, Film T3 (57.56 ± 0.05) and Film T4 (56.14 ± 0.27) had the lowest values, with significant differences between them. High L* values are characteristic of white materials [56]. Therefore, the films evaluated in this study presented a dark brown coloration, as shown in Figure 3.

Finally, the color difference values (ΔE*) showed statistically significant differences (*p* < 0.05). In addition, Films T2 and T3 (10.31 ± 0.01 and 9.56 ± 0.01, respectively) had values greater than 5, indicating that the color difference in these films can be perceived with the eye [57]. In addition, the thermoforming process of the films was accompanied by high temperatures (150 °C), which could have led to the caramelization of residual sugars in the polymeric matrix [1,58].

### 3.5. Thickness and Water Vapor Permeability (WVP)

The thickness of the films obtained is shown in Table 3. The film thicknesses did not show statistically significant differences (*p* > 0.05). These results suggest that the films were homogeneous and that the manufacturing process was standardized. Thickness is a crucial parameter when evaluating barrier properties, as generally, the greater the thickness, the lower the permeability. Water vapor permeability (WVP) is a barrier property of packaging that determines its ability to allow water vapor to pass through per unit area and time. This parameter was evaluated for the films, and the results are shown in Table 3.

The WVP values showed significant variation, ranging from 4.63 to 11.06 × 10^−10^ g·m^−1^·s^−1^·Pa^−1^ (Table 3). This variable was not influenced by thickness, but it was affected by the degree of substitution of the added polysaccharides (*p* < 0.05). Films T1 (control) and T2 showed no statistically significant differences in their WVP values (10.64 × 10^−10^ g·m^−1^·s^−1^·Pa^−1^ and 11.06 × 10^−10^ g·m^−1^·s^−1^·Pa^−1^, respectively) (*p* > 0.05). On the other hand, compared to Films T1 and T2, Films T3 (7.63 × 10^−10^ g·m^−1^·s^−1^·Pa^−1^) and T4 (4.63 × 10^−10^ g·m^−1^·s^−1^·Pa^−1^) recorded the lowest values for WVP (*p* < 0.05). Acetylation of the reinforcing materials (MCC and starch) was performed to decrease the water affinity of the films. In this sense, Film T4 presented the lowest WVP value compared to the other films (Films T1–T3), which is a desirable property for delaying food deterioration. The results of this research showed similar behavior to that observed in a study conducted with acetylated sorghum starch films, in which the WVP dropped from 2.45 × 10^−9^ g·m^−1^·s^−1^·Pa^−1^ to 1.10 × 10^−9^ g·m^−1^·s^−1^·Pa^−1^ in the control (film with native starch) and the film with the highest DS (0.05), respectively [53]. Similarly, the addition of hydrophobic essential oils lowers WVP values. In this regard, Song et al. [59] added different concentrations of lemon essential oil (0.5–2%) to corn and wheat starch films. The authors observed that the WVP of the film without essential oil (3.68 × 10^−10^ g·m^−1^·s^−1^·Pa^−1^) decreased by up to 16% when 2% (*w*/*w*) lemon essential oil was added to the formulation. In general, the WVP results in Table 3 show a behavior similar to that observed for moisture content, water solubility (Table 2), and contact angle (Figure 2), indicating a change in the surface properties of the films as their hydrophobic character increased with the acetylation time.

### 3.6. Mechanical Properties

The mechanical properties of tensile strength (TS), modulus of elasticity (EM), and elongation at break (%E) of the different films are shown in Table 3. The analysis of these properties is of interest, as they can determine the application of the films. Additionally, modification of the polysaccharides can alter the mechanical properties [7]. As for TS, Films T1 and T2 had the lowest value (0.84 ± 0.02 MPa), while Films T3 (1.09 ± 0.01 MPa) and T4 (1.34 ± 0.01 MPa) had the highest values, with significant differences between the treatments. These results agree with those reported by several authors [21,52,54], who have attributed this behavior to greater interaction within the polymeric matrix as the DS of the polysaccharides increases. Colussi et al. [52] observed that the TS increased from 2.48 MPa to 3.22 MPa when native rice starch was replaced by acetylated starch (0.72 DS).

Similar to the trend observed for TS, the %E values increased with the DS of the reinforcement materials. Films T1 and T2 had the lowest elongation (14.16 ± 0.17%), whereas Films 3 (18.35 ± 0.18%) and 4 (21.66 ± 0.71%) showed significantly higher values (*p* < 0.05). These results agree with those reported by Colussi et al. [52] for acetylated rice starch films. These authors report that the %E values, compared with the native starch film (11.35%), were higher when the DS in the starches were 0.38 (95.40%) and 0.45 (109.05%). These results suggest that acetylation results in materials with greater flexibility and elasticity. This behavior is because acetylation generates strong mechanical crosslinking. Additionally, the plasticizer helps reduce the formation of rigid bonds and favors starch-microcellulose-glycerol interactions, thereby forming bonds with greater mobility [60].

Finally, the EM presented a similar behavior to TF. The lowest results (*p* < 0.05) were observed in Film T1 (0.96 ± 0.03 MPa), while in Film T2 it increased significantly to 1.36 ± 0.05 MPa and subsequently to 1.61 ± 0.4 MPa in Film 3. No statistical differences were observed between Film 3 and Film 4 (1.65 ± 0.04 MPa). This behavior can be attributed to acetylation, which exposes active hydroxyl groups on cellulose and starch, thereby promoting mechanical crosslinking within the polymer matrix [21,61]. However, excessive fiber addition can reduce the mechanical properties of the films due to poor distribution or agglomeration, preventing the entire film surface from being covered [62].

### 3.7. Morphological Properties

**Figure 4:**
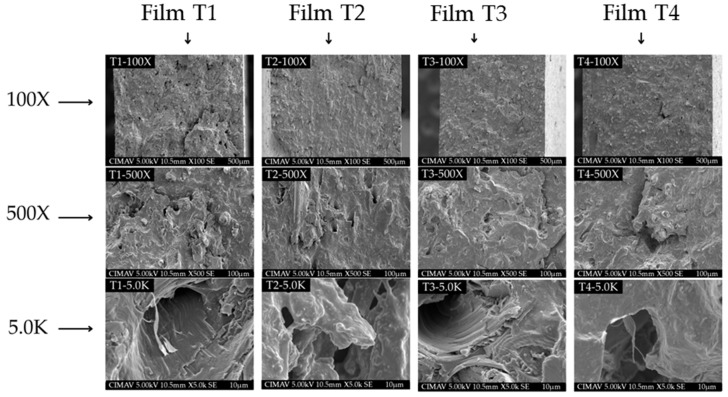
Scanning electron microscopy (SEM) of the films produced with PSI, OEO, and acetylated polysaccharides. Film T1 = film with non-acetylated polysaccharides; Film T2 = film with MCC acetylated for 1 h and starch acetylated for 1 h; Film T3 = film with MCC acetylated for 3 h and starch acetylated for 2 h; Film T4 = film with MCC acetylated for 5 h and starch acetylated for 3 h.

The SEM images of the analyzed samples are shown in Figure 4 at different magnifications. A homogeneous structure is observed across all four treatments, indicating that the extrusion and thermoforming processes are effective for producing films from PSI mixed with starch and MCC. However, as can be seen in Figure 4 at 100× magnification, Films T2–T4 (T2-100×-T4-100×) presented a more compact structure compared to Film T1 (T1-100×). This behavior is similar to that reported by El Halal et al. [51] in biodegradable films of acetylated barley starch added with cellulose fibers from barley hull. The authors noted that in the control treatment (without acetylation), some microcracks were observed; however, these discontinuities were not observed in films with acetylated starch. As the magnification increased, some discontinuities became visible. However, microcracks were more easily seen in the control treatment (Film T1) at magnifications of 500× and 5000× (T1-500× and T1-5.0K). These results suggest that acetylated starch and microcellulose are more compatible with PSI [14,55]. On the other hand, incorporating essential oils into polymer matrices can introduce structural discontinuities due to partial loss through volatilization during production [59,63]. For example, Chen et al. [64] produced films based on starch and poly(butylene adipate-co-terephthalate) containing cinnamon essential oil. The authors reported the presence of discontinuities and micropores attributed to partial volatilization of the essential oil during the extrusion process (155–140 °C). In our study, some of the OEO may have volatilized during extrusion and thermoforming, leading to discontinuities. However, no evident phase separation was observed in the micrographs, suggesting that the OEO is homogeneously dispersed within the polymer matrix. This uniform distribution is likely to reduce the volatilization of the active compounds and facilitate their release, thus maintaining functionality in the films [65]. However, the absence of visible oil droplets in the micrographs could also be due to their partial evaporation during cryofracturing [59]. According to the micrographs of the films evaluated in our study, the compatibility between the polymers is high and improved when the reinforcing materials were acetylated. These observations support the results observed in the evaluation of its mechanical and barrier properties (Table 3).

### 3.8. Thermal Properties

**Figure 5:**
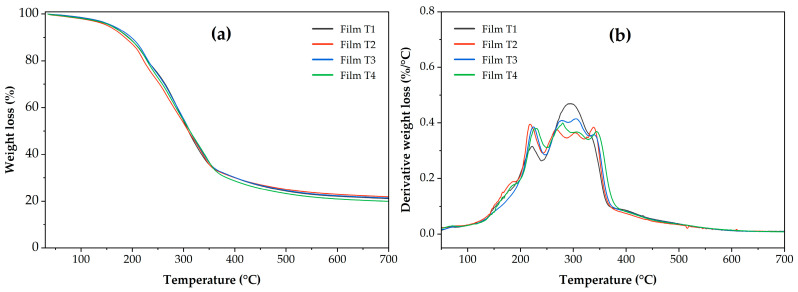
(**a**) Thermogravimetric analysis (TGA) and (**b**) Derivative of thermogravimetric analysis (DTGA) of the films produced with PSI, OEO, and acetylated polysaccharides. Film T1 = film with non-acetylated polysaccharides; Film T2 = film with MCC acetylated for 1 h and starch acetylated for 1 h; Film T3 = film with MCC acetylated for 3 h and starch acetylated for 2 h; Film T4 = film with MCC acetylated for 5 h and starch acetylated for 3 h.

Figure 5 shows the thermal behavior of weight loss as a function of temperature (TGA, Figure 5a) and the derivatives of this behavior (DTGA, Figure 5b). The curves of the derivatives of weight loss as a function of temperature (Figure 5b) reveal the stages of film decomposition. Thermal decomposition of the films occurred in five stages. The first occurred between 80 and 140 °C and was attributed to the loss of moisture present in the samples [66]. The second stage was recorded between 140 and 250 °C, which can be attributed to the loss of water bound and absorbed in the films through hydroxyl groups and the plasticizer present in the polymeric matrix [21,67]. The third stage ranged from 250 to 330 °C, attributed to the decomposition of cellulose and, probably, to compounds of the oregano essential oil bound in the films [9,68]. As shown in Figure 5b, in the third stage, Film T1 exhibited a single peak with greater intensity than the other films. At this stage, Films T2–T4 exhibit two degradation peaks, which can also be attributed to the degradation of the acetyl groups or the degradation of the internal structure of the MCC [19,69]. A fourth stage occurred between 330 and 390 °C, which has been linked to starch degradation. Figure 5b shows that this fourth stage is slightly visible in Film T1; this may be attributed to the greater thermal stability of acetylated starches compared to unacetylated starch (Films T2–T4) [70,71]. Finally, a fifth stage is observed between 390 and 450 °C, attributed to PSI degradation [72,73].

Otherwise, from the TGA thermograms in Figure 5, the most representative thermal variables of the thermal events observed during the sample analysis were determined (Table 4). Regarding weight loss associated with the moisture removed at 100 °C (MC100), Films T3 and T4 had the lowest values (*p* < 0.05), with no statistically significant difference between the treatments. The highest values (*p* < 0.05) were found in Films T1 and T2, with no statistically significant difference between them. This behavior suggests that films containing polysaccharides with higher DS exhibit lower moisture retention [74]. Regarding the temperature at which 10% of the sample is lost (T_90_), Film T4 (198.22 ± 1.07 °C) exhibited the highest thermal stability (*p* < 0.05). This behavior can be attributed to the presence of acetyl groups in acetylated polysaccharides (starch and microcellulose) [48,74,75]. A similar behavior was observed for the temperature at which weight loss begins (T_onset_), with Film T3 and Film T4 being the most thermally stable. Finally, the residual mass at 700 °C (RM700) showed no statistically significant differences between the films (*p* > 0.05).

On the other hand, regarding the DSC analysis, the melting temperature (T_m_) and the change in melting enthalpy (ΔH_m_) are shown in Table 4. Film T1 (213.22 ± 0.06 °C) had the lowest value in T_m_ (*p* < 0.05), while Films T2, T3, and T4 had the highest value without showing statistically significant differences (*p* > 0.05) between them. These results can be attributed to the presence of acetyl groups, which have been reported to alter intermolecular interactions, thereby increasing the energy required to reach the melting state and suggesting greater stability at high temperatures [18,76,77]. The presence of microcellulose is also a factor to consider, as Díaz-Cruz, et al. [78] report that the inclusion of cellulose nanocrystals promotes an increase in the thermal stability of films made from acetylated corn starch with chitosan (T_m_ = 144.9–154 °C). In the present study, stability can be attributed to the acetylation of both reinforcing materials (starch and microcellulose). On the other hand, HΔ_m_ dropped significantly with increased acetylation time, with Film T1 showing the highest value, followed by Film T2, which is statistically different (*p* < 0.05), and finally Films T3 and T4, for which there is no statistical difference (*p* > 0.05). This decrease can be attributed to the incorporation of acetyl groups, which alter the original structure of the polysaccharides, leading to an increase in the amorphous region and a decrease in HΔ_m_ for treatments with longer acetylation times [68,76,79].

### 3.9. Antioxidant Activity

The antioxidant activity of the films, as determined by the DPPH and ABTS methods, is presented in Table 5. As shown in the results, there were no significant differences (*p* > 0.05) in antioxidant capacity between the two methods. This behavior was expected, suggesting that acetylation level did not affect OEO release, as OEO was added at a constant concentration (3.5% *w*/*w*) across all treatments.

High temperatures can affect antioxidant capacity. In this sense, Bodea et al. [80] mention that the antioxidant activity of OEO was 60 mg of ET/100 g per DPPH. In this study, lower values (14.0–14.74 mg ET/100 g) were observed, likely due to the extrusion and thermoforming processes conducted above 100 °C. In addition, the antioxidant capacity of plant extracts and EOs depends mainly on the botanical source and its concentration in the films [81].

### 3.10. Antibacterial Activity

The antibacterial activity of the films produced is shown in Table 5. For *S. aureus*, the largest inhibition halos (*p* < 0.05) were observed in the positive control T0 (2.1 ± 0.15 mm) and Film T3 (1.7 ± 0.12 mm), with no statistically significant difference between them (*p* > 0.05). However, Film T3 did not show statistically significant differences (*p* > 0.05) compared with Films T1, T2 and T4. Similarly, for *E. coli*, the largest inhibition halos were observed in T0 (2.4 ± 0.15 mm), while the lowest values were found in the remaining treatments (T1–T4, 1.7 ± 0.03–1.4 ± 0.00 mm), with no significant difference between them (*p* < 0.05). These differences can be attributed to extrusion and compression molding, as these processes are carried out at high temperatures (125 °C and 150 °C, respectively), at which various compounds can partially degrade, potentially promoting a decrease in microbial activity [68]. Regarding the thermal stability of OEO, the degradation of its volatile compounds begins between 100 and 180 °C, and its complete degradation can occur between 200 and 250 °C [63]. The above suggests that part of the OEO may be lost during extrusion (125 °C) and thermoforming process (150 °C). In this regard, Llana-Ruiz-Cabello et al. [82] obtained active polypropylene films with OEO at nominal concentrations of 3, 5 and 8% (*w*/*w*) by means of an extrusion process at 200–205 °C. The authors measured OEO content in the films and observed an average retention of 80% relative to the theoretical content.

## 4. Conclusions

This study demonstrated the feasibility of obtaining active films from polysuccinimide by adding OEO, starch, and fibers. FTIR and SEM studies demonstrated high compatibility among the materials used in the film formulation, indicating that the acetylation process facilitated their integration. Better component integration, driven by a higher acetyl group content in the polysaccharides (MMC and starch), led to films with superior mechanical properties compared to the control (film without acetylated materials). Furthermore, moisture content, solubility, and WVP decreased with increasing DS in the polysaccharides. Barrier properties are crucial when evaluating a packaging, as low WVP values are generally desired to extend the shelf life of foods. Thermal properties were also improved, as the incorporation of acetylated materials increased thermal stability. Regarding antioxidant and antibacterial activity, all films can be considered active packaging, and increasing the degree of material substitution did not influence OEO release. However, it should be noted that extrusion and thermoforming processes may reduce the concentration of active ingredients in the polymer matrix. Overall, the results suggest that the design of active packaging based on PSI with other polysaccharides has significant potential for food packaging applications.

## Figures and Tables

**Table 1:** Formulation of the films.

Film Code	Component (%, *w*/*w*)				
	PSI	MCC (Acetylation Time)	Starch (Acetylation Time)	Glycerol	OEO
T1 (control)	22	22 (0 h)	22 (0 h)	30.5	3.5
T2	22	22 (1 h)	22 (1 h)	30.5	3.5
T3	22	22 (3 h)	22 (2 h)	30.5	3.5
T4	22	22 (5 h)	22 (3 h)	30.5	3.5

PSI: polysuccinimide, OEO: Oregano essential oil, MCC: microcellulose. Film T1 = film with non-acetylated polysaccharides; Film T2 = film with MCC acetylated for 1 h and starch acetylated for 1 h; Film T3 = film with MCC acetylated for 3 h and starch acetylated for 2 h; Film T4 = film with MCC acetylated for 5 h and starch acetylated for 3 h.

**Table 2:** Moisture content, water solubility and color of the films produced with PSI, OEO, and acetylated polysaccharides.

Film	Moisture (%)	Solubility (%)	L*	a*	b*	ΔE*
T1	13.17 ± 0.08 ^a^	45.64 ± 0.15 ^a^	60.70 ± 0.06 ^a^	9.21 ± 0.21 ^c^	4.34 ± 0.03 ^d^	-
T2	13.50 ± 0.08 ^a^	44.52 ± 0.07 ^a^	60.24 ± 0.28 ^a^	9.88 ± 0.60 ^c^	6.83 ± 0.07 ^c^	10.31 ± 0.01 ^a^
T3	11.44 ± 0.15 ^b^	41.56 ± 0.08 ^b^	57.56 ± 0.05 ^b^	11.65 ± 0.03 ^b^	8.43 ± 0.01 ^b^	9.56 ± 0.01 ^b^
T4	9.81 ± 0.14 ^c^	38.75 ± 0.50 ^c^	56.14 ± 0.27 ^c^	17.17 ± 0.32 ^a^	13.24 ± 0.12 ^a^	4.49 ± 0.09 ^c^

Values represent the mean *±* standard error. Means with different superscript letters (a–d) in the same column indicate statistically significant differences (*p* < 0.05). Film T1 = film with non-acetylated polysaccharides; Film T2 = film with MCC acetylated for 1 h and starch acetylated for 1 h; Film T3 = film with MCC acetylated for 3 h and starch acetylated for 2 h; Film T4 = film with MCC acetylated for 5 h and starch acetylated for 3 h.

**Table 3:** Thickness, water vapor permeability, and mechanical properties of the films produced with PSI, OEO, and acetylated polysaccharides.

Film	Thickness (mm)	WVP × 10^−10^ (g·m^−1^·s^−1^·Pa^−1^)	TS (MPa)	%E	EM (MPa)
Film T1	1.08 ± 0.02 ^a^	10.64 ± 0.14 ^a^	0.86 ± 0.03 ^c^	14.16 ± 0.17 ^c^	0.96 ± 0.03 ^c^
Film T2	1.05 ± 0.02 ^a^	11.06 ± 0.63 ^a^	0.84 ± 0.02 ^c^	15.13 ± 0.17 ^c^	1.36 ± 0.05 ^b^
Film T3	1.06 ± 0.02 ^a^	7.63 ± 0.19 ^b^	1.09 ± 0.01 ^b^	18.35 ± 0.18 ^b^	1.61 ± 0.02 ^a^
Film T4	1.07 ± 0.02 ^a^	4.63 ± 0.08 ^c^	1.34 ± 0.01 ^a^	21.66 ± 0.71 ^a^	1.65 ± 0.04 ^a^

Values represent the mean ± standard error. Means with different superscript letters (a–c) in the same column indicate statistically significant differences (*p* < 0.05). Film T1 = film with non-acetylated polysaccharides; Film T2 = film with MCC acetylated for 1 h and starch acetylated for 1 h; Film T3 = film with MCC acetylated for 3 h and starch acetylated for 2 h; Film T4 = film with MCC acetylated for 5 h and starch acetylated for 3 h.

**Table 4:** Thermal parameters of the films evaluated by TGA and DSC analysis.

Film	TGA				DSC	
	MC100 (%)	T_90_ (°C)	T_onset_ (°C)	RM700 (%)	T_m_ (°C)	HΔ_m_ (J/g)
T1	1.95 ± 0.01 ^a^	188.40 ± 1.77 ^b^	142.13 ± 0.03 ^b^	21.09 ± 0.40 ^b^	213.22 ± 0.06 ^b^	150.89 ± 0.06 ^a^
T2	1.87 ± 0.26 ^a^	189.90 ± 2.81 ^b^	143.10 ± 0.57 ^b^	21.93 ± 1.17 ^a^	216.55 ± 0.43 ^a^	137.45 ± 0.06 ^b^
T3	1.52 ± 0.02 ^b^	191.48 ± 0.84 ^b^	147.58 ± 0.51 ^a^	21.38 ± 0.57 ^a^	218.17 ± 0.49 ^a^	99.83 ± 0.06 ^c^
T4	1.51 ± 0.51 ^b^	198.22 ± 1.07 ^a^	148.60 ± 0.40 ^a^	19.91 ± 2.17 ^a^	216.93 ± 0.93 ^a^	97.83 ± 0.86 ^c^

Values represent the mean ± standard error. Means with different superscript letters (a–c) in the same column indicate statistically significant differences (*p* < 0.05). Film T1 = film with non-acetylated polysaccharides; Film T2 = film with MCC acetylated for 1 h and starch acetylated for 1 h; Film T3 = film with MCC acetylated for 3 h and starch acetylated for 2 h; Film T4 = film with MCC acetylated for 5 h and starch acetylated for 3 h.

**Table 5:** Antioxidant and antibacterial properties of the films produced with PSI, OEO, and acetylated polysaccharides.

Film	Antioxidant Activity		Antibacterial Activity	
	DPPH (mg TE/100 g)	ABTS (mg TE/100 g)	*S. aureus* (mm)	*E. coli* (mm)
T0	------	------	2.10 ± 0.15 ^a^	2.40 ± 0.32 ^a^
T1	14.00 ± 0.44 ^a^	46.65 ± 0.10 ^a^	1.60 ± 0.08 ^b^	1.70 ± 0.03 ^b^
T2	14.38 ± 0.58 ^a^	46.52 ± 0.29 ^a^	1.50 ± 0.12 ^b^	1.60 ± 0.03 ^b^
T3	13.74 ± 0.06 ^a^	46.96 ± 0.09 ^a^	1.70 ± 0.12 ^ab^	1.60 ± 0.18 ^b^
T4	14.31 ± 0.42 ^a^	46.81 ± 0.12 ^a^	1.50 ± 0.05 ^b^	1.40 ± 0.00 ^b^

Values represent the mean ± standard error. Means with different superscript letters (a–b) in the same column indicate statistically significant differences (*p* < 0.05). T0: positive control (paper circle with 3% OEO, *w*/*w*) Film T1 = film with non-acetylated polysaccharides; Film T2 = film with MCC acetylated for 1 h and starch acetylated for 1 h; Film T3 = film with MCC acetylated for 3 h and starch acetylated for 2 h; Film T4 = film with MCC acetylated for 5 h and starch acetylated for 3 h.

## Data Availability

The original contributions presented in this study are included in the article. Further inquiries can be directed to the corresponding author(s).
